# TACC3 promotes colorectal cancer tumourigenesis and correlates with poor prognosis

**DOI:** 10.18632/oncotarget.9628

**Published:** 2016-05-26

**Authors:** Yong Du, Lili Liu, Chenliang Wang, Bohua Kuang, Shumei Yan, Aijun Zhou, Chuangyu Wen, Junxiong Chen, Yue Wu, Xiangling Yang, Guokai Feng, Bin Liu, Aikichi Iwamoto, Musheng Zeng, Jianping Wang, Xing Zhang, Huanliang Liu

**Affiliations:** ^1^ Guangdong Provincial Key Laboratory of Colorectal and Pelvic Floor Diseases, Guangdong Institute of Gastroenterology and The Sixth Affiliated Hospital, Sun Yat-sen University, Guangzhou, Guangdong, China; ^2^ State Key Laboratory of Oncology in South China, Collaborative Innovation Center for Cancer Medicine, Department of Experimental Research, Sun Yat-sen University Cancer Center, Guangzhou, Guangdong, China; ^3^ State Key Laboratory of Oncology in South China, Collaborative Innovation Center for Cancer Medicine, Departments of Pathology and Endoscopy, Sun Yat-sen University Cancer Center, Guangzhou, Guangdong, China; ^4^ Department of Clinical Laboratory, The Sixth Affiliated Hospital, Sun Yat-sen University, Guangzhou, Guangdong, China; ^5^ Institute of Human Virology and Key Laboratory of Tropical Disease Control of Ministry of Education, Sun Yat-sen University, Guangzhou, Guangdong, China; ^6^ State Key Laboratory of Oncology in South China, Collaborative Innovation Center for Cancer Medicine, Sun Yat-sen University Cancer Center, Guangzhou, Guangdong, China; ^7^ Department of Emergency, The Affiliated Nanhua Hospital, University of South China, Hengyang, Hunan, China; ^8^ Division of Infectious Diseases, Advanced Clinical Research Center, Institute of Medical Science, University of Tokyo, Tokyo, Japan; ^9^ Current affiliation: Japan Agency for Medical Research and Development (AMED), Tokyo, Japan

**Keywords:** colorectal cancer, TACC3, proliferation, migration, invasion

## Abstract

Colorectal carcinoma (CRC) is a malignant epithelial tumour with tremendous invasion and metastatic capacity. Transforming acidic coiled-coil protein-3 (TACC3), a frequently aberrantly expressed oncogene, is an important biomarker in various human cancers. Our study aimed to investigate the expression and function of TACC3 in human CRC. We found that TACC3 was over-expressed at both the mRNA and protein levels in CRC cells and in biopsies of CRC tissues compared with normal controls as determined by qRT-PCR, western blot and immunohistochemical (IHC) staining assays. IHC staining of samples from 161 patients with CRC also revealed that TACC3 expression was significantly correlated with clinical stage (*P* = 0.045), T classification (*P* = 0.029) and M classification (*P* = 0.020). Multivariate analysis indicated that high TACC3 expression was an independent prognostic marker for CRC. Patients who had high TACC3 expression had significantly poorer overall survival (OS, *P* = 0.023) and disease-free survival (DFS, *P* = 0.019) compared to patients who had low TACC3 expression. Furthermore, TACC3 knockdown attenuated CRC cell proliferation, colony formation capability, migration and invasion capability, and tumourigenesis in nude mice; these properties were measured using a real-time cell analyser (RTCA), clonogenicity analysis, and transwell and xenograft assays, respectively. These data indicate that TACC3 promotes CRC progression and could be an independent prognostic factor and a potential therapeutic target for CRC.

## INTRODUCTION

Colorectal cancer (CRC) is the third most commonly diagnosed cancer in males and the second in females, being responsible for approximately 1.4 million cases and 0.7 million deaths in 2012 [1]. Tumour markers, such as somatic mutations in BRAF and KRAS, have been used to characterize CRC epidemiology and to guide clinical decision-making. However, activating mutations in BRAF and KRAS only occur in approximately 5-15% and 30-45% of colorectal tumours, respectively [2–4]. Thus, new biomarkers must be identified to further characterize BRAF or KRAS mutation-negative colorectal tumours.

Transforming acidic coiled-coil protein-3 (TACC3), a member of the TACC family, localizes to centrosomes and associates with microtubules [5–7]. TACC3 is essential for haematopoietic stem cell function [8] and plays a role in meiotic progression in bovine oocytes [9]. TACC3 was also reported to act as a negative regulator of Notch signalling through binding to CDC10/Ankyrin repeats [5]. High TACC3 expression is correlated with ovarian cancer [10], glioblastoma [11], oesophageal squamous cell carcinoma [12], hepatocellular carcinoma [13, 14], gastric carcinoma [15] and non-small cell lung cancer [16]. The referenced studies have revealed that TACC3 mainly promotes tumour progression by enhancing cell proliferation, cancer stem cell populations and cancer cell migration [12, 14]. In several types of tumours, a common TACC3 fusion gene known as FGFR3-TAAC3 has been shown to promote the development of cancer cells by promoting cell proliferation [17–19].

To identify new biomarkers and potential therapeutic targets for CRC, we analysed CRC microarray data from the Oncomine database and identified TACC3 as a potential gene for these purposes. In the current study, we investigated the correlation between TACC3 expression and CRC prognosis as well as the function of TACC3 in the development of CRC.

## RESULTS

### TACC3 is over-expressed in CRC

**Figure 1:**
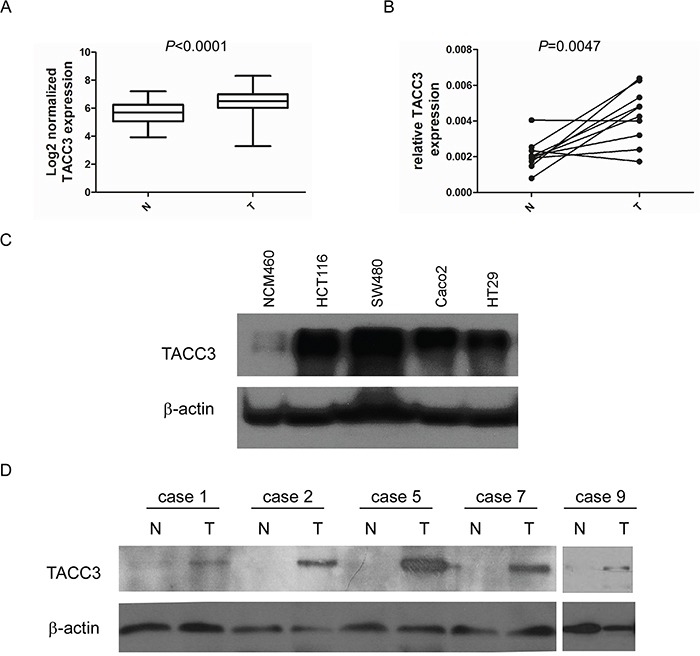
TACC3 is over-expressed in CRC **A.** Log2-normalized expression of TACC3 in TCGA data collected from the Oncomine database. **B.** mRNA expression of TACC3 in 10 pairs of CRC and adjacent normal tissues determined by q-PCR. **C.** Protein expression of TACC3 in CRC and normal cell lines determined by western blotting. **D.** Protein expression of TACC3 in 5 pairs of representative CRC and adjacent normal tissues determined by western blotting.

**Figure 2:**
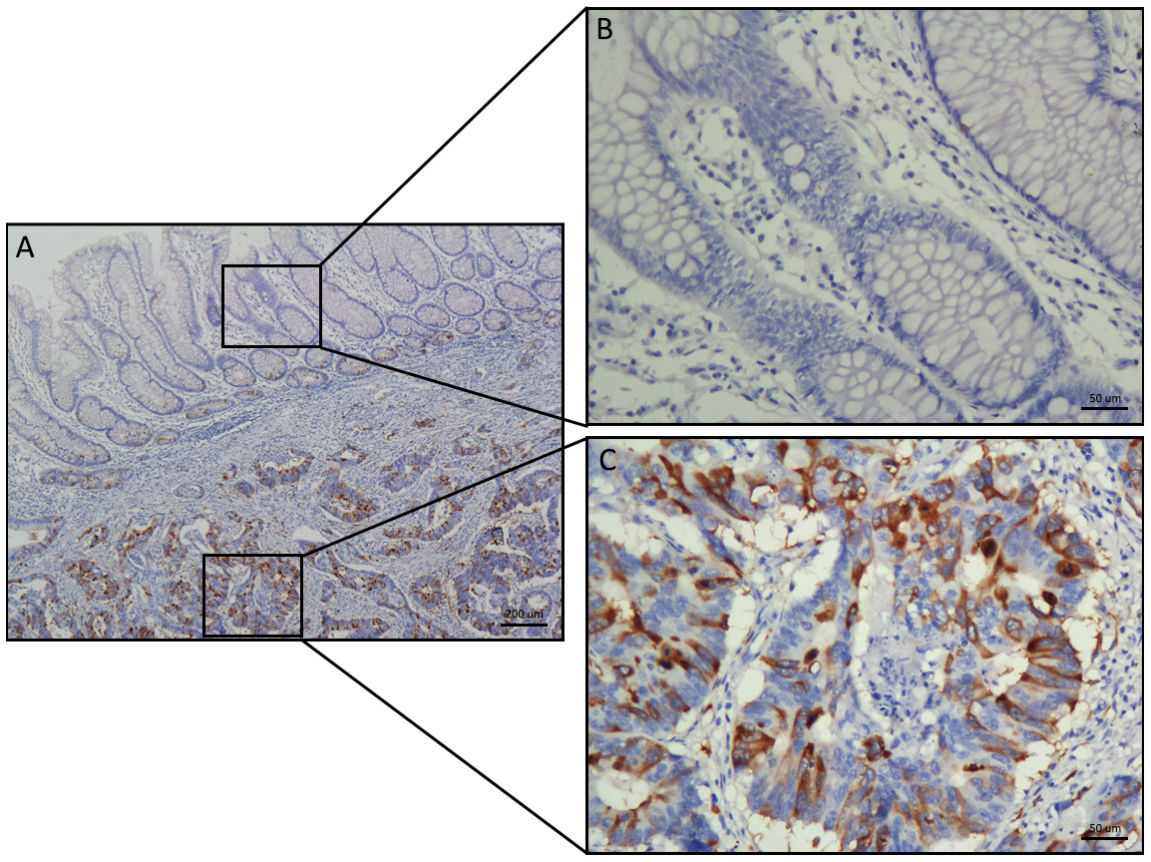
Comparative analysis of TACC3 expression in CRC and adjacent normal tissues by IHC staining **A.** TACC3 staining in typical specimens of CRC and adjacent normal tissues. **B.** Negative or weak TACC3 staining was observed in the cytoplasm of tumour-adjacent normal tissues. **C.** Strong TACC3 expression was observed in the cytoplasm of CRC tissues. (Left panel: magnification 40×; right panel: magnification 200×).

To identify a new biomarker and potential treatment target for CRC, we analysed transcript expression microarray data from the Oncomine database (GSE20916; N = 40, T = 96) and identified a differentially expressed gene called TACC3 that was over-expressed in CRC samples compared with normal controls (*P* < 0.001) (Figure 1A). We confirmed this result by measuring the mRNA and protein expression of TACC3 in cancerous and normal fresh tissues collected from patients with CRC. We found that both the mRNA and protein levels of TACC3 were increased in the CRC samples compared with normal control samples (Figure 1B, 1D). Similarly, we found that TACC3 was highly expressed in the CRC cell lines HCT116, SW480, Caco2 and HT29 but was weakly expressed in the human colonic epithelial cell line NCM460 (Figure 1C). In addition, this result was further confirmed by IHC staining, which showed strong expression of TACC3 in cancer cells (Figure 2A, 2C) and negative or weak expression in adjacent normal cells (Figure 2A, 2B) from the same patient specimens.

### Relationships between TACC3 expression and clinicopathological variables

**Figure 3:**
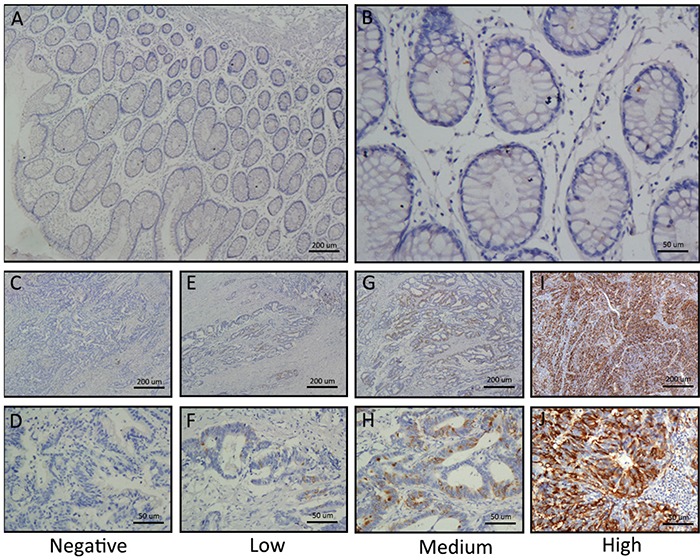
Differential expression of TACC3 in CRC tissues shown by IHC staining **A** and **B.** Negative TACC3 staining in normal colorectal tissues (negative control); (A) 40×, (B) 200×. **C** and **D.** Negative TACC3 staining in CRC tissue; (C) 40×, (D) 200×. **E** and **F.** Weak TACC3 staining in cytoplasm; (E) 40×, (F) 200×. **G** and **H.** Moderate TACC3 staining in cytoplasm; (G) 40×, (H) 200×. **I** and **J.** Strong TACC3 staining in cytoplasm; (I) 40×, (J) 200×.

To determine whether TACC3 expression is correlated with the clinical progression of CRC, 161 CRC and corresponding adjacent colorectal epithelial tissues were collected (Supplementary Table S1), and IHC staining with a TACC3 antibody was performed. Our results showed that TACC3 localized to the cytoplasm (Figure 2C). Tumours with an immunoreactivity score (IRS) exceeding 5 were classified as having high TACC3 expression. As a result, 77 (47.8%) tumour samples had high TACC3 expression, and 84 (52.2%) tumour samples had low TACC3 expression (Table 1). Data corresponding to the tumours with negative (Figure 3C, 3D), low (Figure 3E, 3F), medium (Figure 3G, 3H) and high (Figure 3I, 3J) TACC3 staining are presented in Figure 3. However, none of the corresponding adjacent normal tissue presented high TACC3 expression (Figure 2B, Figure 3A, 3B). TACC3 expression in CRC was correlated with clinical stage (*P =* 0.045), T classification (*P =* 0.029) and M classification (*P =* 0.020) (Table 1). However, TACC3 expression was not correlated with age, gender, N classification, pathologic differentiation, histological type or location (Table 1).

**Table 1:** Correlation of clinicopathological parameters and TACC3 expression

Variable	TACC3 expression			
	All cases	Low expression	High expression	*P* value^a^
**Age (years)^b^**				0.546
< 53	63	31 (49.2%)	32 (50.8%)	
≥ 53	98	53 (54.1%)	45 (45.9%)	
**Gender**				0.989
Female	71	37 (52.1%)	34 (47.9%)	
Male	90	47 (52.2%)	43 (47.8%)	
**Clinical Stage**				**0.045**
I+II	65	40 (61.5%)	25 (38.5%)	
III	69	35 (50.7%)	34 (49.3%)	
IV	27	9 (33.3%)	18 (66.7%)	
**T classification**				**0.029**
T1+T2	33	14 (42.4%)	19 (57.6%)	
T3	40	28 (70%)	12 (30%)	
T4	88	42 (47.7%)	46 (52.3%)	
**N classification**				0.447
N0	71	41 (57.7%)	30 (42.3%)	
N+				
**M classification**				**0.020**
M0	133	75 (56.4%)	58 (43.6%)	
M1	28	9 (32.1%)	19 (67.9%)	
**Pathologic Differentiation**				0.549
Well	3	1 (33.3%)	2 (66.7%)	
Moderately	150	80 (53.3%)	70 (46.7%)	
Poorly	8	3 (37.5%)	5 (62.5%)	
**Histological Types**				0.865
Non-mucinous adenocarcinoma	147	77 (52.3%)	70 (47.7%)	
mucinous adenocarcinoma	14	7 (50.0%)	7 (50.0%)	
**Location**				0.267
Colon	93	52 (55.9%)	41 (44.1%)	
Rectal	68	32 (47.1%)	36 (52.9%)	
**Vital status**				0.058
Alive	108	62 (57.4%)	46 (42.6%)	
Death	53	22 (41.5%)	31 (58.5%)	

a Chi-square test;

b Median age.

### Correlation between TACC3 expression and survival outcomes

**Figure 4:**
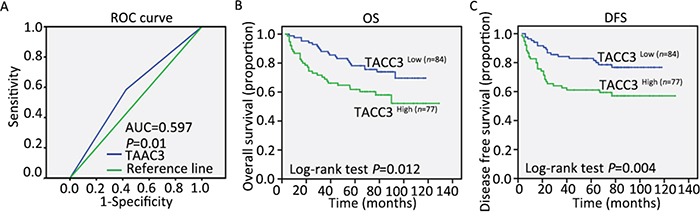
Correlation between TACC3 expression and survival outcomes **A.** The sensitivity and 1-specificity of TACC3 expression in CRC tissues were plotted. The area under the curve (AUC) and the p value as calculated by ROC curve analysis are indicated (AUC = 0.597, *P =* 0.01). The five-year overall survival (OS) rate was 50.7% in the 161 patients with CRC. **B** and **C.** High TACC3 expression was significantly correlated with OS (*P* = 0.012) and DFS (*P =* 0.004) in all of the included patients with TACC3.

To determine an optimal cut-off value to differentiate low TACC3 expression, a ROC curve was utilized according to the results of IHC staining analysis (Figure 4A). The ROC curve for clinical stage possessed the smallest distance (0.0, 1.0), indicating that TACC3 expression has the greatest prognostic ability (maximum sensitivity and specificity) for clinical stage (ROC-2) (Supplementary Figure 1). Therefore, an IRS score of 5 was chosen as a cut-off value for low TACC3 expression. We analysed the correlation between TACC3 expression and the survival of patients with CRC using Kaplan-Meier methodology (log-rank test). Survival data were available for all 161 patients. In multivariate survival analysis, the patients with a low level of TACC3 expression demonstrated a significantly better OS (Figure 4B, *P =* 0.023) and DFS (Figure 4C, *P =* 0.019) than those with a high level of TACC3 expression. The multivariate survival analysis suggested that T classification (HR = 1.469, 95%CI 1.050-2.054, *P =* 0.025), M classification (HR = 3.893, 95%CI 1.177-12.874, *P =* 0.026) and TACC3 expression (HR = 1.095, 95%CI 1.095-3.314, *P =* 0.023) were independent predictors of OS (Table 2). However, only M classification (HR = 4.319, 95%CI 1.395-13.374, *P =* 0.011) and TACC3 expression (HR = 2.045, 95%CI 1.125-3.719, *P =* 0.019) were independent predictors of DFS (Table 2). These data demonstrated that the expression level of TACC3 had a significant association with the clinicopathological characteristics of the patients. Overall, TACC3 appears to be a potential prognostic predictive factor in CRC.

**Table 2:** Univariate and multivariate analyses of clinicopathological and TACC3 expression for overall and diseasefree survival in overall cohort (n = 161) by Cox regression analyses

Variables	Univariate analysis		Multivariate analysis	
	HR (95% CI)	*P* value	HR (95% CI)	*P* value
**Overall survival**				
Age (< 53 vs. ≥ 53 years)	0.673 (0.392-1.155)	0.151		
Gender (female vs. male)	0.814 (0.475-1.395)	0.454		
Clinical stage (I-II vs. III vs. IV)	2.351 (1.745-3.166)	**0.000**	0.933 (0.397-2.194)	**0.873**
T clasffication (I-II vs. III vs. IV)	1.790 (1.274-2.514)	**0.001**	1.469 (1.050-2.054)	**0.025**
N clasffication (N0 vs. N+)	5.001 (2.500-10.003)	**0.000**	3.544 (0.758-16.566)	0.108
M clasffication (yes vs. no)	5.380 (3.020-9.584)	**0.000**	3.893 (1.177-12.874)	**0.026**
Tumor differentiation (I vs. II vs. III)	1.436 (0.571-3.611)	0.442		
Histological type (no vs. yes)	1.032 (0.410-2.594)	0.947		
Location (colon vs. rectal)	1.386 (0.808-2.379)	0.236		
TACC3 expression (low vs. high)	1.994 (1.153-3.448)	**0.012**	1.095 (1.095-3.314)	**0.023**
				
**Disease-free survival**				
Age (< 53 vs. ≥ 53 years)	0.615 (0.349-1.084)	0.093		
Gender (female vs. male)	0.884 (0.502-1.558)	0.670		
Clinical stage (I-II vs. III vs. IV)	3.077 (2.141-4.421)	**0.000**	1.512 (0.792-2.887)	**0.210**
T clasffication (I-II vs. III vs. IV)	1.490 (1.094-2.028)	**0.011**	1.274 (0.933-1.741)	**0.127**
N clasffication (N0 vs. N+)	3.331 (1.725-6.429)	**0.000**	1.168 (0.461-2.956)	**0.744**
M clasffication (yes vs. no)	10.35(5.766-18.575)	**0.000**	4.319 (1.395-13.374)	**0.011**
Tumor differentiation (I vs. II vs. III)	1.178 (0.430-3.230)	0.750		
Histological type (no vs. yes)	0.639 (0.199-2.057)	0.453		
Location (colon vs. rectal)	1.665 (0.944-2.938)	0.078		
TACC3 expression (low vs. high)	2.301 (1.281-4.131)	**0.004**	2.045 (1.125-3.719)	**0.019**

Abbreviations: HR, hazard ratio; CI, confidence interval.

### Knockdown of TACC3 attenuates proliferation and colony formation capability in CRC cell lines

**Figure 5:**
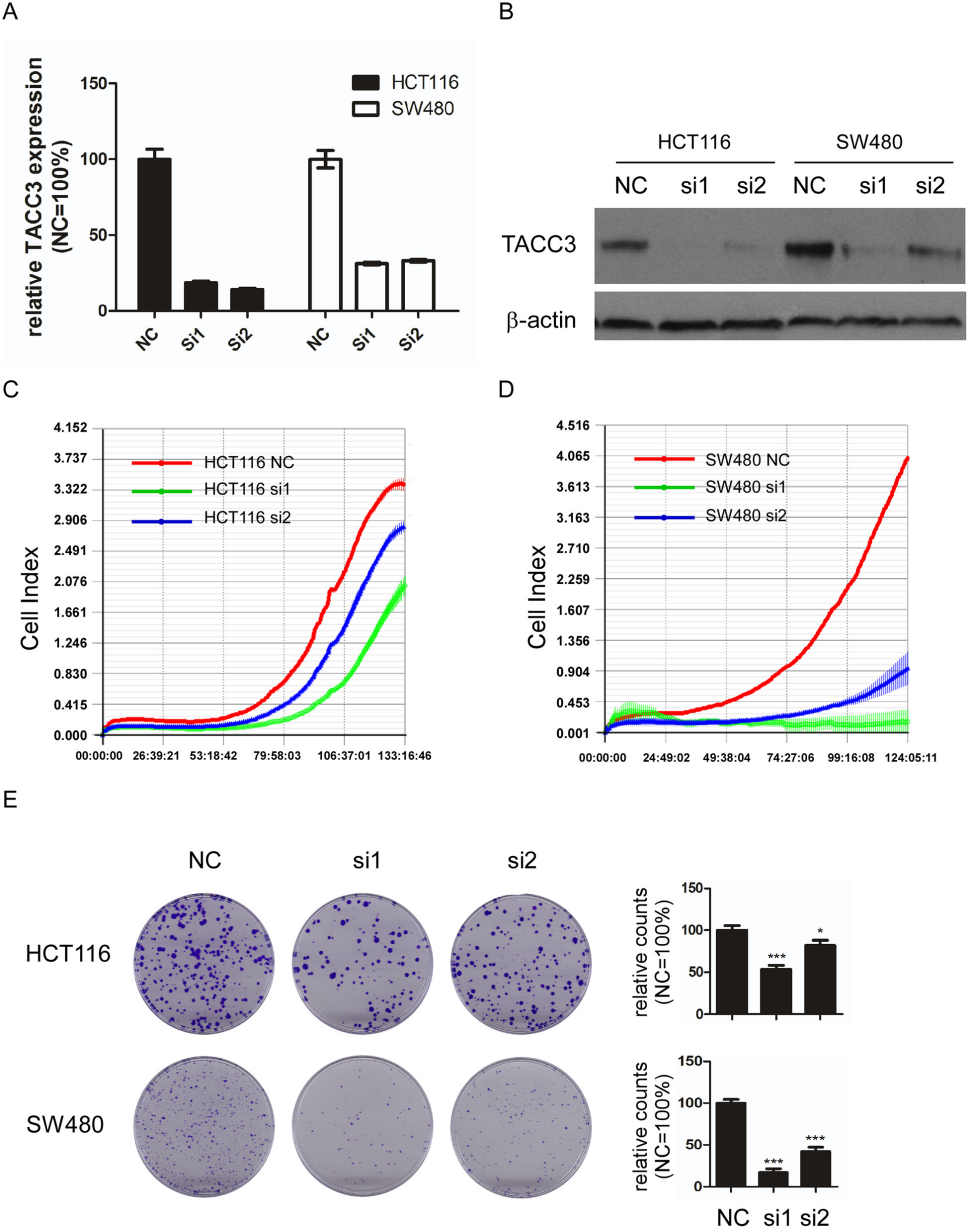
Knockdown of TACC3 attenuates proliferation and colony formation capability in CRC cell lines **A.** TACC3 mRNA expression after siRNA-mediated knockdown in HCT116 and SW480 cells. **B.** TACC3 protein expression after siRNA-mediated knockdown in HCT116 and SW480 cells. **C** and **D.** Knockdown of TACC3 attenuates the proliferation of HCT116 (C) and SW480 (D) cells as measured by RTCA. **E.** Knockdown of TACC3 significantly attenuates colony formation capability in HCT116 and SW480 cells as measured by colony formation assays. The data are presented as the mean ± SD (*P < 0.05, ***P < 0.001). NC, negative control; si1, siTACC3-1; si2, siTACC3-2.

As shown in Table 2, TACC3 expression was correlated with T classification in the patients with CRC. Thus, we investigated the potential roles of TACC3 in CRC tumourigenesis using a real-time cell analyser (RTCA) assay and colony formation assay after knockdown of TACC3 expression through siRNA transfection. As shown in Figure 5, TACC3 siRNA transfection significantly decreased the expression of TACC3 in the HCT116 and SW480 cell lines both at the mRNA (Figure 5A) and protein (Figure 5B) levels. In addition, the knockdown of TACC3 significantly decreased the proliferation (Figure 5C, 5D) and the colony formation numbers (Figure 5E) of the assessed CRC cell lines. These results suggest that TACC3 has a crucial role in promoting CRC cell growth.

### Knockdown of TACC3 inhibits migration and invasion capability in CRC cell lines

**Figure 6:**
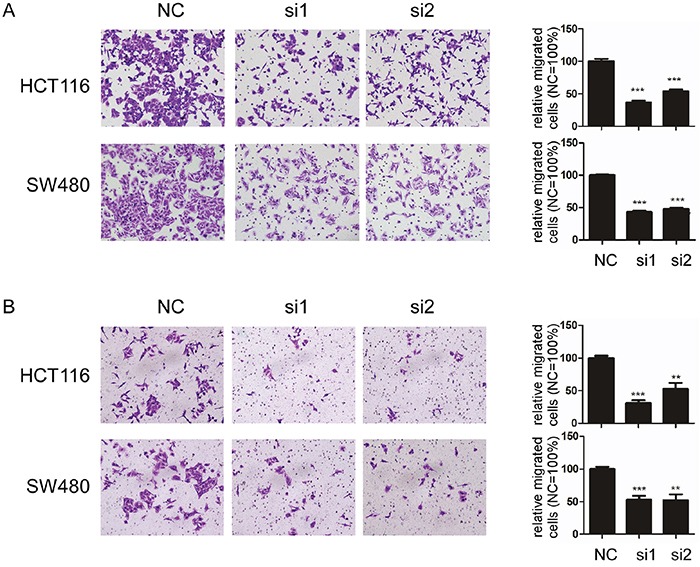
Knockdown of TACC3 inhibits migration and attenuates invasion capability in CRC cell lines **A** and **B.** Knockdown of TACC3 significantly inhibits migration (A) and attenuates invasion (B) capability in HCT116 and SW480 cells. C. EMT marker expression after knockdown of TACC3 in CRC cells as determined by western blotting with β-actin as an internal control. The data are presented as the mean ± SD (**P < 0.01, ***P < 0.001). NC, negative control; si1, siTACC3-1; si2, siTACC3-2.

As shown in Table 2, TACC3 expression was correlated with M classification in the patients with CRC. Thus, we investigated the potential roles of TACC3 in CRC migration and invasion using transwell assays after knockdown of TACC3 expression through siRNA transfection. As shown in Figure 6, the knockdown of TACC3 significantly decreased the migration (Figure 6A) and invasion (Figure 6B) capabilities of the HCT116 and SW480 cell lines. These data demonstrated that TACC3 could promote CRC migration and invasion.

### Knockdown of TACC3 inhibits CRC tumourigenesis in nude mice

**Figure 7:**
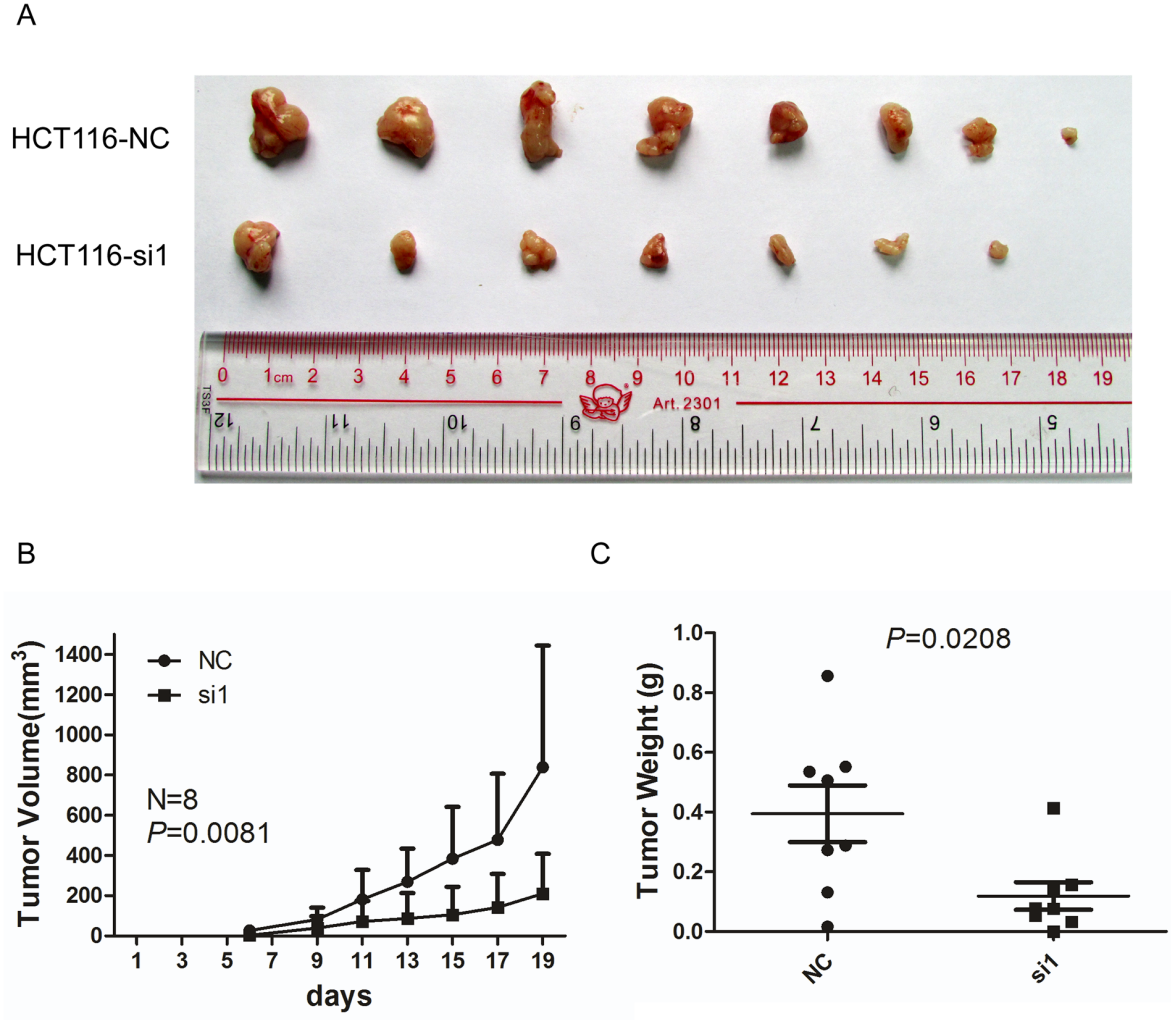
Knockdown of TACC3 inhibits tumour growth in nude mice **A.** Images of tumours formed by HCT116 cells transfected with NC or si1. **B.** The growth curves of the tumours formed by HCT116 cells transfected with NC or si1. The data are presented as the mean ± SD (N = 8 mice per group). **C.** Weight differences in tumours formed by HCT116 cells transfected with NC or si1 and injected in nude mice. The data are presented as the mean ± SD (N = 8 mice per group). NC, negative control; si1, siTACC3-1.

To further analyse the role of TACC3 in tumourigenesis *in vivo*, HCT116 cells that were treated with TACC3 siRNA were subcutaneously injected into 5-week-old NOD/SCID female mice. Tumour formation was assessed after 6 days. The repression of TACC3 significantly reduced the volumes (Figure 7A, 7B, *P =* 0.0081) and weights (Figure 7A, 7C, *P =* 0.028) of the xenografts. In summary, our data indicate that knockdown of TACC3 repressed tumour growth in nude mice.

## DISCUSSION

At present, surgical resection remains the main therapy used for patients with CRC [20]. However, patients with the same TNM stage experience different curative effects associated with resection [20]. Therefore, additional predictive factors of prognosis are needed to better evaluate therapy outcomes and help develop individualized treatment plans. Several methods have been used to identify new biomarkers for CRC, including translational approaches investigating perturbations in gene expression or genomic instability, which can be identified using animal models of CRC [21], and high-throughput technologies such as massive parallel sequencing [22]. Bioinformatics methods combined with public cancer databases also have been widely used to find potential biomarkers. In the current study, Oncomine data analysis and q-PCR, western blotting and IHC analyses revealed increased levels of TACC3 in CRC tissues compared with adjacent non-malignant tissues. These results demonstrated that TACC3 could be a potential biomarker for CRC.

Correlation analysis of clinicopathological parameters and TACC3 expression revealed that TACC3 expression was significantly correlated with clinical stage (*P =* 0.045), T classification (*P =* 0.029) and M classification (*P =* 0.020) but was not significantly correlated with age, gender, N classification, pathologic differentiation, histological type or location. Cox multivariate analysis showed that increased TACC3 expression correlated with T classification and M classification and a shorter DFS and OS. These results suggest that CRC patients with high TACC3 expression tend to have a poorer prognosis, which is similar to previous reports on different cancers other than CRC [10, 12–16]. Thus, patients with a high level of TACC3 expression could be recommended to receive aggressive radiotherapy and chemotherapy to reduce tumour malignancy and metastasis. Collectively, our data indicate that TACC3 is a potentially independent and predictive factor for the survival of patients with CRC.

Our results demonstrated that knock down of TACC3 reduced CRC cell proliferation, clonogenicity, migration and invasion capability, as well as tumour growth in nude mice. These data suggested that TACC3 may be associated with tumour growth and metastasis in CRC patients, which was consistent with the observation that TACC3 expression was correlated with T and M classification. Accumulating evidence indicated that using RNAi based approaches to influence molecular pathways had the potential of being translated into cancer therapeutic applications. Given that there is no data on TACC3 RNAi based research for treating CRC, novel therapeutic strategies targeting TACC3 related pathways could be explored in future study. A recent study revealed suppression of TACC3 activates the p38-p53-p21 stress signalling pathway [23], which is consistent with our observations (data not shown). However, TACC3 promotes epithelial-mesenchymal transition (EMT), which had been reported in osteogenic sarcoma [24] and cervical cancer [25], haven't been observed in this study (data not shown). Considering TACC3 might promote tumourigenesis in a tissue-specific manner, the mechanism of how TACC3 influences CRC progression need to be further studied.

In conclusion, TACC3 expression as detected by IHC could serve as an independent predictor of patient survival for patients with CRC, and TACC3 should be explored as a potential therapeutic target for CRC.

## MATERIALS AND METHODS

### Clinical samples

The patients enrolled in this study were diagnosed with CRC and underwent curative resection between 1999 and 2007 at the Sun Yat-sen University Cancer Center (SYSUCC). None of the patients had received radiotherapy or chemotherapy prior to surgery. A total of 161 qualified CRC tissue samples and adjacent para-cancerous tissue samples were sectioned and subjected to IHC staining. Diagnosis and histological differentiation were evaluated according to the World Health Organisation classification, and tumour staging was based on the American Joint Committee on Cancer TNM staging system. The patients' clinical data were collected from hospital records after surgery. This study was approved by the research ethics committee of SYSUCC.

In Figure 1A, the TACC3 mRNA expression levels in 134 colorectal samples from the NCBI Dataset GSE20916 are shown (N = 44, T = 90); these were measured using TACC3 microarray probe set number 218308_at.

### Cell culture

The HCT116, SW480, Caco2, HT29 and NCM460 cell lines were cultured in RPMI 1640 (Invitrogen) supplemented with 10% foetal bovine serum (FBS; Hyclone, Logan, UT) in a humidified 5% CO_2_ incubator at 37°C.

### Immunohistochemistry

In total, 161 CRC specimens were subjected to immunohistochemical (IHC) staining to visualize the expression of TACC3. The specimens were deparaffinized and rehydrated. After three washes in PBS, the specimen slides were boiled in a high-pressure cooker for 2.5 min in an EDTA (pH 8.0) buffer to retrieve antigen. Then, 5% BSA was used to block non-specific binding. After blocking, the sections were incubated with a rabbit anti-human monoclonal antibody against TACC3 (1:400 dilution; ab134154, Abcam) in blocking buffer at 4°C overnight in a moist chamber. Blocking buffer lacking the primary antibody was used as a negative control. Then, the sections were incubated with horseradish peroxidase for 30 minutes at 37°C, followed by incubation with 3,30-diaminobenzidine solution (4 min) for visualization.

The staining results were scored based on two criteria: (1) the proportion of positive tumour cells in the tumour tissue, rated as 0 (0%), 1 (1% to 10%), 2 (11% to 50%), 3 (51% to 75%), or 4 (76% to 100%), and (2) the intensity of the staining, rated as 0, absent; 1, weak; 2, moderate; and 3, strong.

### Selection of cut-off score

We selected a cut-off score for the patients with CRC who had low TACC3 expression by using the 0,1-criterion in receiver operating characteristic (ROC) curve analysis. For the TACC3 score, the sensitivities and specificities of select clinicopathological variables were plotted, thus generating various ROC curves. The count was selected as the cut-off value that was closest to the point that had maximum sensitivity and specificity. The specimens defined as having high TACC3 expression were those with scores above or equal to the cut-off value, while the specimens with low TACC3 expression represented those with scores below the value. To perform ROC curve analysis, some clinicopathological variables were dichotomized, including clinical stage (I-II vs. III vs. IV), T classification (I-II vs. III vs. IV), N classification (N0 vs. N+), and M classification (yes vs. no).

### Quantitative real-time RT-PCR

Quantitative real-time RT-PCR (q-PCR) was performed using an ABI 7500 Sequence Detection System. The primer sequences are listed below.

TACC3:

S: CCTCTTCAAGCGTTTTGAGAAAC, AS: GCCCTCCTGGGTGATCCTT;

ATCB:

S: AGCCTCGCCTTTGCCGATCC, AS: ACATGCCGGAGCCGTTGTCG.

### Western blotting

We conducted western blotting following a previously described method [26]. Briefly, equal amounts of protein were separated by 8% SDS-PAGE and electrophoretically transferred onto PVDF membranes. A rabbit anti-human TACC3 antibody (1:1000) and β-actin antibody (1:4000; ProteinTech, China) were used to detect the expression of TACC3 and β-actin. β-Actin was used as an internal control.

### siRNA knockdown assay

TACC3 siRNAs were obtained from Ray Biotech, Inc. (Guangzhou, China). In total, 1.5×10^5^ HCT116 or SW480 cells were seeded into 6-well plates, and 24 h later, the cells were transfected with the TACC3 siRNAs using RNAiMAX transfection reagents (Invitrogen) according to the manufacturer's instructions. The target sequences of the TACC3 siRNAs were as follows:

siTACC3-1 (si1): CCACAGATCTGAACTCCAT and

siTACC3-2 (si2): GGATTACCTGGAGCAGTTT.

### Real-time cell analyser assay

To analyse CRC cell proliferation, a real-time cell analyser (RTCA) assay was performed using an xCELLigence RTCA system (Roche Applied Sciences, Indianapolis, IN), which consists of a RTCA impedance analyser, an SP station, 96-well E-Plates, and a computer with RTCA software to control the operation of the system. For each RTCA assay, 50 μl of growth medium was added to each well for the impedance background measurement. Then, 150 μl of RPMI 1640 medium supplemented with 10% FBS and containing 2000 cells was added to each well, and cell growth was assessed for approximately 5 days.

### Colony formation assay

Cells (500-1000 cells per well) were evenly seeded in 6-well plates and cultured for 7-14 days. After fixation with methanol for 10 min, the colonies were stained with 0.5% crystal violet in 20% methanol and counted. All of the experiments were performed independently in triplicate.

### Transwell migration and invasion assays

Migration assays were performed using 24-well transwell plates (Corning) with an 8-μm pore size polycarbonate filter. In total, 5×10^4^ HCT116 cells or 1×10^5^ SW480 cells were seeded onto the upper surfaces of the filters. The cells that migrated to the lower surfaces of the filters were photographed at a magnification of 200× after 24-48 h, and the total number of migrated cells was counted. Invasion assays were performed using a similar protocol to that employed for the migration assay, and 5% Matrigel was pre-plated in the transwell plates before seeding the cells.

### Nude mouse xenograft assay

To analyse the role of TACC3 in CRC tumour formation *in vivo*, a nude mouse xenograft assay was conducted as previously described [27]. Briefly, 8 female BALB/c nude mice were injected with cells that had been transfected with either TACC3 siRNA or negative control siRNA. The mice were purchased from Beijing Slac Laboratory Animal Co., Ltd (Shanghai, China) and maintained in microisolator cages. All of the mice were randomly assigned to two groups, and each received a subcutaneous injection of 150 μl of a viable cell suspension mixture (5×10^6^) containing a 90% HCT116-NC (negative control) or HCT116-siTACC3-1 (si1) cell suspension and 10% Matrigel. When the tumours could be palpated, tumour size was measured using callipers every other day. Tumour volume was calculated using the formula L×W^2^×0.5, where L is the largest dimension and W is the perpendicular diameter. All of the mice were sacrificed on the third week after injection, and the individual tumours were weighed.

### Statistical analysis

Statistical analysis was performed using the SPSS 17.0 statistical software package. The ROC curve used to define the TACC3 IRS cut-off value was analysed using the MedCalc statistical software package, version 11.0.1 (MedCalc Software bvba, Ostend, Belgium). Correlations between TACC3 expression and clinicopathologic variables were evaluated using Pearson's χ2 test. Overall survival (OS) and disease-free survival (DFS) curves were acquired using the Kaplan-Meier method and compared with the log-rank test. A Cox proportional hazard model was used to conduct the multivariate survival analysis shown in Table 2 (criteria *P* < 0.05). Analyses of differences between groups (in the RTCA, migration and nude mice xenograft assays) were achieved using a two-tailed t test. A two-sided *P* < 0.05 was considered statistically significant.

## SUPPLEMENTARY FIGURE AND TABLE


