# Supplementary material for: TACC3 promotes colorectal cancer tumourigenesis and correlates with poor prognosis
Source: Oncotarget. 2016 May 26;7(27):41885–97. doi: 10.18632/oncotarget.9628 (PMC5173103; doi:10.18632/oncotarget.9628)
Supplement: Supplementary file 1 [file oncotarget-07-41885-s001.pdf]

TACC3 promotes colorectal cancer tumourigenesis and correlates with poor prognosis

SUPPLEMENTARY FIGURE AND TABLE

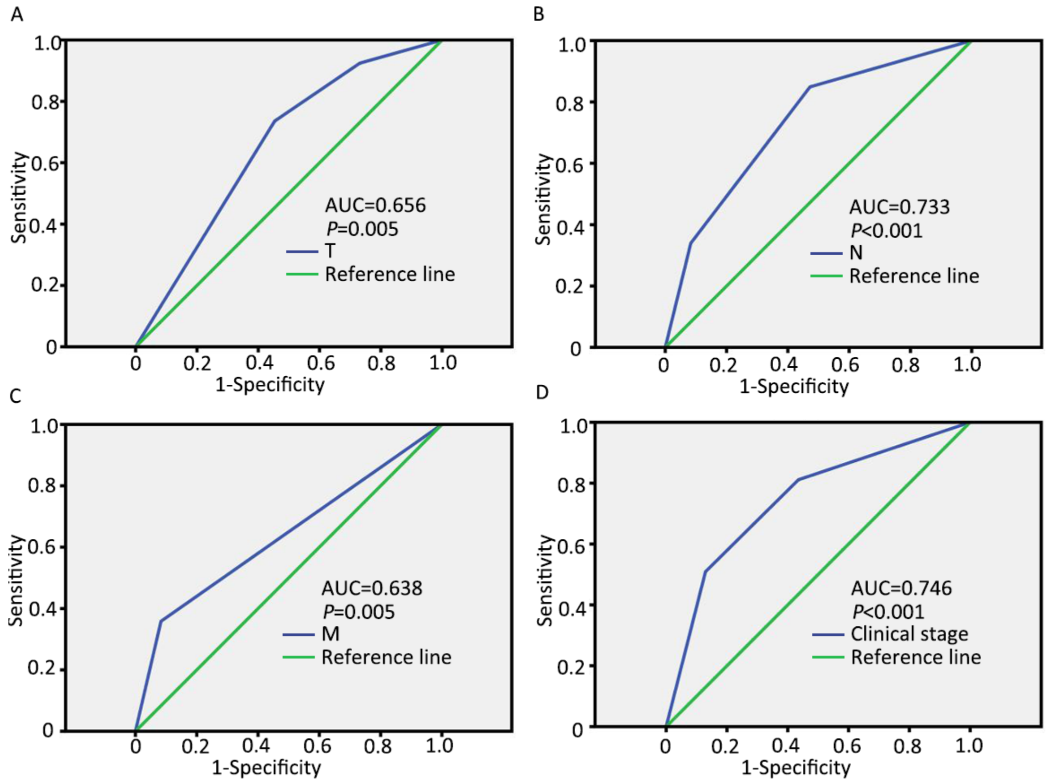

Supplementary Figure S1: ROC-2 analysis. The sensitivity and 1-specificity for several variables in CRC patients, including T classification **A.**, N classification **B.**, M classification **C.**, and clinical stage **D.**, were plotted. AUC, area under the curve.

Supplementary Table S1: Clinicopathologic characteristics of CRC patients (n=161)

Characteristics	No.(%)
**Age (years)[b]**	53.3±11.8
**Gender**	
Female	71(44.1)
Male	90(55.9)
**Clinical Stage**	
I+II	65(40.4)
III	69(42.9)
IV	27 (16.7)
T classification	
T1+T2	33(20.5)
T3	40(24.8)
T4	88(54.7)
**N classification**	
N0	71(44.1)
N+	90(55.9)
**M classification**	
M0	133(82.6)
M1	28(17.4)
**Pathologic Differentiation**	
Well	3(1.9)
Moderately	150(93.2)
Poorly	8(4.9)
**Histological Types**	
Non-mucinous adenocarcinoma	147(91.3)
mucinous adenocarcinoma	14(8.7)
**Location**	
Colon	93(57.8)
Rectal	68(42.2)
**Vital status**	
Alive	108(67.1)
Death	53(32.9)
